# Heme Oxygenase 1 (HO-1) as an Inhibitor of Trafficking of Normal and Malignant Hematopoietic Stem Cells – Clinical and Translational Implications

**DOI:** 10.1007/s12015-020-10083-w

**Published:** 2020-11-16

**Authors:** Mariusz Z. Ratajczak, Mateusz Adamiak, Janina Ratajczak, Magda Kucia

**Affiliations:** 1 Stem Cell Institute at James Graham Brown Cancer Center, University of Louisville, 500 S. Floyd Street, Rm. 107, Louisville, KY 40202 USA; 2 Department of Regenerative Medicine, Center for Preclinical Research and Technology, Medical University of Warsaw, Warsaw, Poland

**Keywords:** Stem cell mobilization, stem cell homing, SDF-1, S1P, Heme oxygenase 1, HO-1, Chemotaxis, Adhesion, Hematopoietic recovery, Hematopoietic stem cells, Leukemia

## Abstract

Evidence indicates that bone marrow (BM)-residing hematopoietic stem/progenitor cells (HSPCs) are released into peripheral blood (PB) after administration of pro-mobilizing drugs, which induce a state of sterile inflammation in the BM microenvironment. In the reverse process, as seen after hematopoietic transplantation, intravenously injected HSPCs home and engraft into BM niches. Here again, conditioning for transplantation by myeloablative chemo- or radiotherapy induces a state of sterile inflammation that promotes HSPC seeding to BM stem cell niches. Therefore, the trafficking of HSPCs and their progeny, including granulocytes and monocytes/macrophages, is regulated by a response to pro-inflammatory stimuli. This responsiveness to inflammatory cues is also preserved after malignant transformation of hematopoietic cells. Results from our laboratory indicate that the responsiveness of hematopoietic cells to pro-inflammatory stimuli is orchestrated by Nlrp3 inflammasome. As reported, HO-1 effectively attenuates intracellular activation of Nlrp3 inflammasome as well as the pro-inflammatory effects of several humoral mediators, including complement cascade (ComC) cleavage fragments that promote migration of hematopoietic cells. Based on this finding, inhibition of HO-1 activity may become a practical strategy to enhance the mobilization and homing of normal HSPCs, and, alternatively, its activation may prevent unwanted spread and in vivo expansion of leukemic cells.

**Figure:**
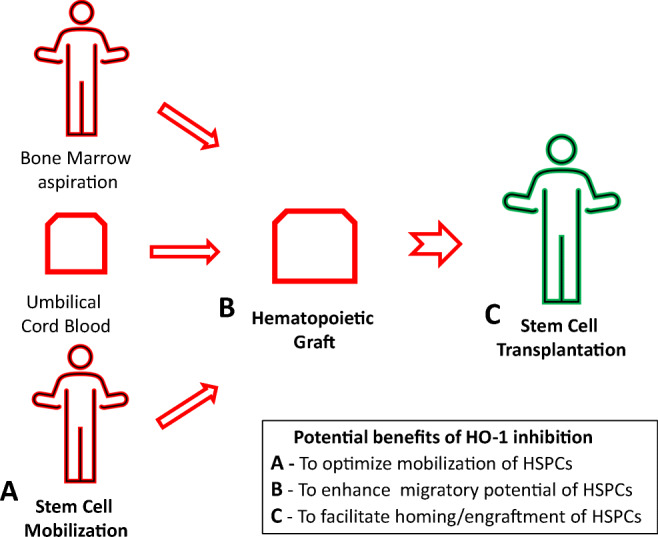
Graphical Abstract

Graphical Abstract

## Introduction

Heme oxygenase is represented by two isozymes, of which heme oxygenase 2 is constitutively expressed, and heme oxygenase 1 (HO-1) is induced in response to its substrate heme and other mediators [1]. This inducible stress-response enzyme not only catalyzes the degradation of heme (e.g., released as a component of hemoglobin from erythrocytes) but also has important pleiotropic functions in various physiological and pathophysiological states associated with cellular stress, infections, and tissue/organ damage [1–5]. Recent studies have implicated HO-1 as an important regulator of mitochondrial biogenesis and mitochondrial function, as well as lipid and polyamines metabolism [6]. There have been several reports indicating its ant-inflammatory potential and negative effects on the migration of neutrophils and monocytes [7, 8]. In support of these findings, bone marrow mononuclear cells (BMMNCs) from mice deficient in HO-1 showed enhanced migration and repopulated lethally irradiated recipients with more rapid kinetics [9].

We propose that mobilization of hematopoietic stem/progenitor cells (HSPCs) occurs in response to sterile inflammation, induced in the bone marrow (BM) microenvironment after administration of pro-mobilizing drugs, such as the cytokine granulocyte colony stimulating factor (G-CSF) and AMD3100 (Plerixafor), a small-molecule antagonist of the chemokine receptor CXCR4 [10–16]. On the other hand, a state of sterile inflammation in BM is also induced during myeloablative conditioning for hematopoietic transplantation by chemo- and/or radiotherapy and plays a role in facilitating homing and engraftment of transplanted cells [16–18]. An important role in all these processes is played by activation of the cellular and humoral arms of innate immunity. Accordingly, pharmacological mobilization of HSPCs requires activation of the cells responsible for innate immunity, including granulocytes, monocytes, and dendritic cells [10, 19, 20]. An important effector of the egress of these cells from BM into PB is the activated complement cascade (ComC), which is supported by the proteolytic activity of the coagulation cascade (CoaC). Mice that lack crucial components of the ComC are deemed poor mobilizers [21], and they display defective BM seeding efficiency or homing of transplanted wild type HSPCs [22, 23].

An important integrating role in the response of innate immunity cells and HSPCs to these pro-inflammatory cues is played by an intracellular pattern-recognition receptor (PPR) known as the NOD-like receptor (NLR) family pyrin domain-containing protein 3 (Nlrp3) inflammasome [17, 24, 25]. This intracellular protein complex recognizes both endogenous danger-associated molecular pattern molecules (DAMPs), such as extracellular ATP (eATP), and certain pathogen-associated molecular pattern molecules (PAMPs) [24, 25]. Its basic expression is primed in innate immunity cells by intestinal Gram-negative bacterial lipopolysaccharide (LPS), and it becomes activated by several DAMPs or alarmines, including the abovementioned eATP [17, 24, 25]. The Nlrp3 inflammasome is also challenged in response to ComC cleavage fragments, such as the anaphylatoxins C3a and C5a as well as the non-lytic form of membrane attack complex (MAC), which is composed of C5b-C9 [17, 24, 25].

What is important for this review is that HO-1 has emerged as an inhibitor of the Nlrp3 inflammasome [26, 27], and this explains its negative effect on the trafficking of hematopoietic cells, which will be discussed below. Thus, inhibition of HO-1 may become a strategy for increasing the mobilization and homing of normal HSPCs [28, 29]. By contrast, its upregulation may prevent unwanted spread in vivo and the expansion of leukemic cells [30, 31].

### The Role of HO-1 in Physiological and Stress-induced Mobilization of HSPCs

To better understand why HSPCs egress from BM into PB, these cells can be regarded as tireless travelers as they migrate during development and then move to where active hematopoiesis occurs. They are at first identified in the blood islands at the bottom of the yolk sac, where primitive hematopoiesis is initiated [32]. Subsequently, they are detected in the hematogenic endothelium of the dorsal aorta and other vessels in the developing fetus. During the second trimester of gestation the fetal liver becomes a major hematopoietic organ in mammals [32]. Finally, at the beginning of the third trimester, HSPCs settle into the developing BM microenvironment. During adult life these cells continuously circulate in PB at a low level in order to maintain a constant level of active hematopoiesis in distant parts of the BM [32, 33]. Another function of their circulation is that these cells “patrol” peripheral tissues for potential infection foci [34]. It has been shown that they may locally differentiate in such places, supplying leukocytes and macrophages in addition to those that originate in BM, to reach infected or damaged tissues.

The number of circulating HSPCs in peripheral blood increases in response to systemic infections, tissue or organ injuries, strenuous exercise, or after administration of the abovementioned pro-mobilizing drugs, such as G-CSF or AMD3100. As proposed, activation of the cellular and humoral arms of innate immunity orchestrates egress of these cells from BM into PB [16, 17].

It is well known that HSPCs are retained in BM niches due to interaction between the CXCR4 chemokine receptor and very late antigen 4 (VLA-4, also known as α_4_β_1_ integrin) receptor, which are expressed on the surface of HSPCs [10–16]. Their respective ligands involved in BM retention, the α-chemokine stromal-derived growth factor 1 (SDF-1) and vascular adhesion molecule 1 (VCAM-1, also known as CD106), are expressed by cells in the BM stem cell niches (e.g., by osteoblasts and stromal fibroblasts) [10–16]. Harvested from PB after pharmacological mobilization, HSPCs are a convenient source of cells for hematopoietic transplantation [13, 14]. We proposed a crucial role for the fifth protein component of the ComC (C5) in the mobilization of HSPCs. C5-deficient mice are poor mobilizers, because C5 cleavage fragments (C5a and C5b) are required for optimal release of these cells from BM into PB [21]. The C5a anaphylatoxin (i) activates neutrophils and monocytes in the BM microenvironment to secrete DAMPS as well as proteolytic and lipolytic enzymes, (ii) permeabilizes the endothelium at the BM–PB barrier, and (iii) chemoattracts neutrophils and monocytes into PB that pave the way for HSPCs to reach the PB. The major chemoattractant for HSPCs in PB is sphingosine-1-phosphate (S1P), which is already at a high level in PB [35–37]. This level can be additionally increased after C5b-C9 complex (MAC)-mediated release from erythrocytes [35].

Since the release of DAMPs from innate immunity cells in response to pro-mobilizing agents or the generation of activated ComC cleavage fragments both trigger mobilization, we became interested in the mechanisms that control this process. Our recent research indicates an important role for the Nlrp3 inflammasome as orchestrator of this process, as it is expressed in the BM microenvironment, in innate immunity cells, and in HSPCs themselves [17]. We also became interested in the potential factors that limit mobilization, as there are relatively limited results bearing on this topic. Inhibitory effects on mobilization have been reported so far mainly for serine protease inhibitors (serpins) and tissue inhibitors of metalloproteinases (TIMPs) [38–40].

Based on the role of ComC-mediated inflammation, the pro-inflammatory effects of its cleavage fragments, C3a and C5a, and the hemolytic activity observed during pharmacological mobilization of HSPCs, we became interested in the potential role of HO-1, an intracellular anti-inflammatory enzyme. This enzyme is known to inhibit ComC-initiated inflammation by upregulating the ComC inhibitors decay-accelerating factor (DAF or CD55) and CD59 on the surface of endothelial cells [41]. On the other hand, HO-1 deficiency in humans and mice predisposes them to injuries in response to stress [9, 42]. Finally, HO-1 directly regulates the expression of a major BM chemoattractant for HSPCs, SDF-1 [43]. To address the role of HO-1 in BM retention and mobilization of HSPCs, we have employed HO-1 knockout mice that were heterozygous (HO-1^+/–^) or homozygous (HO-1^–/–^) [28]. We found that, while these animals at the age of 6–8 weeks still have similar numbers of HSPCs in BM and PB as their wild type (WT) littermates, this similarity diminishes with advancing age, as HO-1 deficiency leads to anemia [28]. We also found that HO-1^–/–^ mice display a reduced SDF-1 mRNA level in BM stromal cells, which implies a negative effect on the retention of HSPCs in BM niches. This finding was subsequently supported by the observation of defective adhesion of wild type HSPCs to BM stromal cells from HO-1^–/–^ mice. Pharmacological mobilization studies employing G-CSF or AMD3100 revealed that HO-1-deficient mice are easy mobilizers, and this effect followed the degree of HO-1 deficiency (HO-1^–/–^ > HO-1^+/–^ > wild type). Our experiments with HO-1-deficient mice were reproduced using a small-molecule inhibitor of HO-1, tin protoporphyrin IX (SnPP) [28]. Moreover, we found that the enhanced mobilization of HSPCs in HO-1-deficient mice correlated with enhanced activation of the ComC [28]. Thus, our studies identified HO-1 as an important negative modulator of HSPC mobilization.

Our recent research indicates an important role for the Nlrp3 inflammasome in HSPC trafficking. It has been reported that this intracellular PRR is negatively regulated by HO-1, as seen for example in LPS-induced acute lung injury and sepsis [26, 27]. Therefore, we hypothesized that a similar effect could occur in HSPCs. To address the role of HO-1 in the inhibition of Nlrp3 inflammasome-mediated trafficking of HSPCs, we exposed these cells to SnPP and observed inhibition of its activation, as measured by release of interleukin-1β from these cells, after exposure to an Nlrp3 inflammasome activation agent, the antibiotic nigericin [44]. Finally, our results showing a pro-mobilizing effect by a nontoxic small-molecule inhibitor of HO-1 (SnPP) suggests that blockade of HO-1 would be a promising strategy in clinical settings to facilitate pharmacological mobilization of these cells for hematopoietic transplantation [28].

### The Role of HO-1 in Homing/engraftment of HSPCs

HSPCs isolated from PB (i) after pharmacological mobilization by leukopheresis, (ii) aspiration from BM, or (iii) harvesting from umbilical cord blood (UCB) are employed for BM reconstitution after hematopoietic transplantation. These cells are infused intravenously into patients, who before transplantation are undergoing myeloablative conditioning by chemo- or radiotherapy, which is aimed to destroy the patient’s pathological hematopoiesis and to empty stem cell niches in BM for newly transplanted HSPCs [13, 14]. After infusion into PB, these cells (i) respond to BM chemoattractants, (ii) navigate towards BM and adhere to BM sinusoid vessels, (iii) cross the PB–BM endothelial barrier, and (iv) settle into hematopoietic niches. Myeloablative conditioning for transplantation also induces a state of sterile inflammation in the BM microenvironment, evidenced by the release of DAMPs from innate immunity cells and activation of the ComC [17, 24, 25]. The Nlrp3 inflammasome becomes activated in response to DAMPs and ComC cleavage fragments, both in migrating HSPCs and in those cells in the BM microenvironment that survived myelobalative treatment (stromal cells, endothelial cells, and osteoblasts) [17, 24, 25]. Therefore, we became interested in the potential role of HO-1 in this process and whether HO-1 could have a negative effect on homing and engraftment.

To address this question, we first employed a small-molecule inhibitor of HO-1 (SnPP) to downregulate HO-1 activity in murine BMMNCs [28]. This inhibition resulted in enhanced chemotaxis of these cells in response to a gradient of SDF-1, which is a principal BM chemoattractant, and to sphingosine-1-phosphate (S1P), which plays a role as a supportive BM chemoattractant. Importantly, SnPP exposure also increased the chemotactic responsiveness of HSPCs. In positive control experiments, we upregulated HO-1 activity in murine BMMNCs by employing an activator of HO-1 (CoPP) and observed the opposite effect on the migration of murine BMMNCs and HSPCs in response to SDF-1 and S1P gradients. In parallel, we obtained similar results with human UCB cells [28, 29].

Next, we performed in vivo homing experiments employing BMMNCs exposed to SnPP before transplantation into lethally irradiated WT animals. Twenty-four hours after transplantation, we observed an increase in the number of these HO-1-inhibited and transplanted cells in BM [29]. Finally, lethally irradiated mice transplanted with BMMNCs that had been previously exposed to SnPP had significantly accelerated recovery of leukocyte and platelet counts in PB [29]. Importantly, we found the opposite effect on the migration of HSPCs when HO-1 was upregulated by the small-molecule activator CoPP.

In support of our results showing a highly migratory state of HSPCs after downregulation of HO-1, another group reported that BMMNCs from mice lacking one HO-1 allele (HO-1^+/–^) showed accelerated hematopoietic recovery from myelotoxic injury, as HO-1^+/–^ HSPCs repopulated lethally irradiated recipients with more rapid kinetics [9]. This could again be explained by the fact that HO-1-deficient HSPCs show enhanced chemotaxis in response to SDF-1 and S1P BM-homing gradients. Nevertheless, HO-1-defient cells in these experiments were ineffective in conferring radioprotection in serial repopulation transplantations.

Summarizing, since HO-1 is a negative regulator of cell migration and downregulates Nlrp3 inflammasomes, transient ex vivo inhibition of HO-1 activity in the HSPCs present in hematopoietic grafts would enhance their homing to BM and accelerate hematopoietic recovery after transplantation [29]. Thus, we propose that this relatively simple strategy of ex vivo exposure of the graft to an HO-1 inhibitor may find practical application in clinical settings, particularly when the number of HSPCs harvested for transplantation is low. This occurs with poor mobilizers of HSPCs, after poor BM harvest by aspiration, and in the case of UCB transplants in which the number of HSPCs in a single UCB unit is limited. Moreover, since the Nlrp3 inflammasome expressed in HSPCs and in the BM microenvironment are crucial for homing and engraftment of HSPCs, and since HO-1 is its inhibitor, downregulation of this anti-inflammatory enzyme promotes trafficking of HSPCs in an Nlrp3 inflammasome-dependent manner.

### The Role of HO-1 in Migration and Systemic Spread of Leukemic Cells

To study the potential effect of HO-1 on migration and adhesion of malignant hematopoietic cells, we established three human hematopoietic cell lines (Raji, K562, and Jurkat) in which HO-1 was overexpressed after transducing cells with an HO-1-encoding vector [29]. Similarly, as in experiments with normal BMMNCs in which HO-1 was upregulated by the small-molecule activator CoPP, HO-1 overexpression was correlated with significant inhibition of the migration of malignant cells in response to SDF-1 and S1P gradients as well as with enhanced adhesion to fibronectin-coated plates. Next, in the converse experiment we successfully downregulated HO-1 expression in these cells by employing an shRNA strategy or exposing them to the HO-1 inhibitor SnPP and observed that downregulation of HO-1 was correlated with enhanced chemotactic responsiveness to SDF-1 and S1P gradients and decreased adhesion to fibronectin-coated plates [29].

As mentioned above, an important role in systemic in vivo trafficking of hematopoietic cells is played by the ComC, which, as a crucial arm of innate immunity and is involved both in mobilization of HSPCs from BM into PB and in their homing to BM. The ComC can also be activated in patients in response to infection or radio- or chemotherapy when applied as a cancer treatment in patients [31]. We asked whether activation of the ComC in leukemic patients enhances the migratory potential of malignant blasts, as this enhanced motility may contribute to the systemic spread of leukemic cells. We found that leukemia cell lines as well as clonogenic blasts isolated from primary chronic myelogenous leukemia (CML) and acute myelogenous leukemia (AML) patients express receptors for C3a and C5a on their cell surfaces and respond by chemotaxis and increased adhesion to stimulation by both ComC cleavage fragments [31]. What is important and relevant to this review, we also found that HO-1 was a negative regulator of ComC-mediated trafficking of leukemic cells. Moreover, stimulation of leukemic cells by C3a or C5a activates p38 MAPK and, as a result of this downregulated HO-1 expression, these cells are rendered more mobile. This result confirmed the involvement of p38 MAPK in regulating the expression of HO-1 [45]. Based on these observations, activation of the ComC in leukemia/lymphoma patients (e.g., as a result of accompanying infections or radio- or chemotherapy treatment) may enhance the unwanted motility of malignant cells and contribute to their spread. Since this occurs in a p38 MAPK–HO-1-dependent manner, inhibition of p38 MAPK or upregulation of HO-1 by small-molecule activators would have a beneficial effect on preventing expansion and systemic spread of leukemia/lymphoma cells when the ComC becomes activated [31].

In support of this prediction, we first downregulated p38 MAPK in human leukemia cell lines (U937 and KG1a) by employing the specific small-molecule inhibitor SB203580, and in its presence neither cell line responded with p38 MAPK activation after stimulation with C3a, C5a, or SDF-1, and, more importantly, downregulation of p38 MAPK resulted in upregulation of HO-1 in these cells [31].

Next, to validate this result in vivo we exposed U937 cells for 2 hours to C3a, C5a, the HO-1 inhibitor SnPP, or the HO-1 activator CoPP and 24 hours after sublethal irradiation to destroy any residual immunity injected these cells intravenously into immunodeficient SCID mice [31]. The mice were subsequently sacrificed 24 hours after i.v. injection of leukemic cells, and the seeding of human malignant cells to bone marrow, lung, and liver was evaluated by RQ-PCR. We detected these cells in murine tissues by performing PCR to amplify human satellite DNA sequences, a method to monitor murine–human chimerism previously described by us.

We observed enhanced spread of malignant cells in immunodeficient mice after exposure of leukemic cells to C3a and C5a as well as to the HO-1 inhibitor SnPP. By contrast, the opposite effect was seen with the downregulation of HO-1 after exposure of leukemic cells to CoPP [31]. Moreover, a similar effect of HO-1 upregulation or downregulation on the spread of leukemic cells in an in vivo immunodeficient SCID mouse model was subsequently confirmed by employing human Raji cells in which HO-1 expression was stably upregulated by employing an HO-1-encoding vector or downregulated using an shRNA strategy [31].

As a follow up to this study, we became interested in the role of bioactive phospholipids in the migration and systemic spread of leukemic cells. We focused on the most important members of this family, including S1P, ceramide-1-phosphate (C1P), lysophosphatidylcholine (LPC), and its derivative lysophosphatidic acid (LPA) [30]. These factors emerged as important mediators regulating the trafficking of normal and cancer cells; however, their role was not well studied in leukemic cells. We employed ten human myeloid and lymphoid cell lines as well as blasts from AML patients and exposed them to S1P, C1P, LPC, or LPA [30]. We found that leukemic cells express functional receptors for these factors and respond to stimulation by phosphorylation of p42/44 MAPK and AKT. Moreover, what is important for this review, all bioactive phospholipids enhanced cell migration and adhesion of leukemic cells by downregulating expression of HO-1 in these cells, in a similar manner as occurs after stimulation with C3a and C5a. HO-1 expression was again downregulated in a p38 MAPK-dependent manner. By contrast, downregulation of p38 MAPK by SB203580 enhanced expression of HO-1, followed by decreased migration of leukemic cells, in vitro and their seeding efficiency to vital organs in vivo after injection into immunodeficient mice. Based on these findings, we proposed that the prometastatic effects of bioactive phospholipids on human leukemic cells occur by downregulating HO-1 in a p38 MAPK-dependent manner, and again inhibitors of p38 MAPK or stimulators of HO-1 activity could find application in inhibiting the spread of leukemic cells [30]. It is important to note that bioactive phospholipids, like the ComC fragments C3a and C5a, are upregulated in patient tissues after treatment with cytostatics or irradiation [31].

We expect that a similar mechanism also operates in patients exposed to chemotherapeutics or irradiation after upregulation of migration-promoting chemokines, cytokines, and growth factors. This, however, requires further study. Thinking about the upregulation of HO-1 expression as a potential anti-metastatic treatment, we have to consider other effects of HO-1 that promote the growth of cancer cells .Specifically, HO-1 reportedly plays a role in cancer progression, and its expression in cancer cells correlates with enhanced tumor growth, metastatic and angiogenic potential, resistance to chemotherapy, tumor escape, and poor prognosis [46]. Thus, upregulation of HO-1 in vivo based on its pro-migratory properties could turn out be a double-edged sword [47].

## Conclusions

**Fig. 1:**
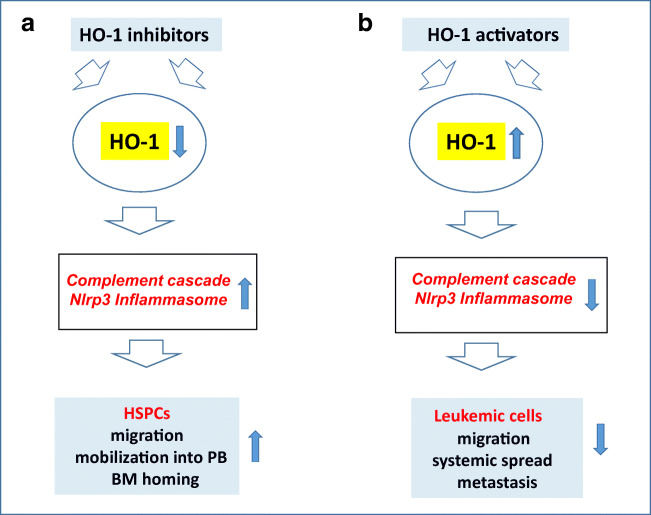
HO-1 regulates the trafficking of normal hematopoietic stem progenitor cells (HSPCs) and leukemic cells. **a** HO-1 is an inhibitor of the complement cascade (ComC) and the Nlrp3 inflammasome, which are both required for optimal mobilization of HSPCs from bone marrow into peripheral blood and their migration, homing, and engraftment after transplantation. It has been found that inhibition of HO-1 promotes all these processes. **b** HO-1 inhibits the trafficking of leukemic cells. Therefore, enhancing the activity of HO-1 in leukemic cells by HO-1 activators may be helpful in preventing their migration and unwanted systemic spread and metastasis

Based on the findings presented, HO-1 may become an important target for strategies to modulate migration of normal HSPCs as well as leukemic cells [28–31]. In the case of normal HSPCs, downregulation of HO-1 by employing small-molecule inhibitors would enhance the mobilization process as well as their homing and engraftment after transplantation [28, 29]. In these applications, HO-1 inhibitors could be employed for better biological effects, perhaps together with “mild” activators of the Nlrp3 inflammasome. As we envision, the ex vivo exposure of HSPCs to an HO-1 inhibitor or an “mild” Nlrp3 inflammasome activator before infusion into patients would enhance their homing and engraftment [17]. It would be particularly important in cases when the number of HSPCs available for transplantation is limited, as when the cells collected by leukopheresis are poor mobilizers, there is a low number of HSPCs aspirated from BM, or there is a low number of HSPCs present in a single unit of UCB [13–15]. Also important to consider, while normal physiological “mild” activation of the Nlrp3 inflammasome is crucial to maintaining the trafficking of HSPCs, its hyperactivation may lead to caspase-1-mediated cell death by pyroptosis, as seen for example during a cytokine storm in COVID19-infected patients or in complications of CAR-T cell therapy [48, 49]. In such cases, HO-1 activators may ameliorate this unwanted effect and protect HSPCs from pyroptotic damage [50, 51]. On the other hand, since HO-1 also inhibits the migration of leukemic cells [30, 31], a potential strategy to upregulate its expression as an anti-metastatic treatment should be weighed against the unwanted tumor growth-promoting effects of this enzyme [46]. Therefore, more research is needed to address this dilemma in appropriate experimental animal models. Finally, in addition to inducible HO-1 it would be interesting to investigate in a future a role of constitutively expressed HO-2 in trafficking of HSPCs. To support this HO-2 deficient mice display magnified inflammation and aberrant neutrophil function that may potentially impact on migration of normal and malignant hematopoietic cells [52] (Fig. 1).

## Abbreviations

BM: bone marrow
BMMNC: bone marrow mononuclear cells
C1P: ceramide 1 phosphate
CoaC: coagulation cascade
ComC: complement cascade
CXCR4: chemokine receptor
DAMPs: danger associated molecular patter molecules
eAdo: extracellular adenosine
eATP: extracellular adenosine triphosphate
HO-1: heme oxygenase − 1
HSPCs: hematopoietic stem/progenitor cells
LPA: lysophosphatidicacid
LPC: lysophosphatidylcholine
LPS: lipopolysaccharide
Mac: membrane attack complex
mPB: mobilized peripheral blood
NLR: nod like receptor
PAMPS: pathogen associated molecular pattern molecules
PB: peripheral blood
SDF-1: stromal derived factor - 1
S1P: sphingosine – 1 phosphate
UCB: umbilical cord blood
VCAM-1: vascular adhesion molecule 1
